# Olive Pomace Phenolic Compounds: From an Agro-Industrial By-Product to a Promising Ocular Surface Protection for Dry Eye Disease

**DOI:** 10.3390/jcm11164703

**Published:** 2022-08-11

**Authors:** Nikolaos Katsinas, Uta Gehlsen, Laura García-Posadas, Soraya Rodríguez-Rojo, Philipp Steven, María J. González-García, Amalia Enríquez-de-Salamanca

**Affiliations:** 1 Institute of Applied Ophthalmobiology (IOBA), University of Valladolid (UVa), Campus Miguel Delibes, Paseo de Belén 17, 47011 Valladolid, Spain; 2 Research Institute on Bioeconomy (BioEcoUVa), High Pressure Processes Group, School of Engineering, University of Valladolid (UVa), Dr. Mergelina Str., 47011 Valladolid, Spain; 3 Biomedical Research Networking Center in Bioengineering, Biomaterials and Nanomedicine (CIBER-BBN), Campus Miguel Delibes, Paseo de Belén 17, 47011 Valladolid, Spain; 4 Department of Ophthalmology, Medical Faculty and University Hospital, University of Cologne, Kerpenerstrasse 62, 50937 Cologne, Germany; 5 Cluster of Excellence: Cellular Stress Response in Aging-Associated Diseases (CECAD), University of Cologne, 50937 Cologne, Germany

**Keywords:** desiccating stress, dry eye syndrome, inflammation, olive pomace phenolic extract, hydroxytyrosol

## Abstract

Dry eye (DED) is a prevalent disease with immune-mediated inflammation as the principal pathophysiological etiology. Olive pomace, the major by-product of the olive oil industry, is rich in high-value polyphenols. Their anti-inflammatory and immunomodulatory activities were determined on human CD4+ T cells (hTCD4+) and in a DED animal model. The viability of hTCD4+ cells isolated from peripheral blood and activated with phytohemagglutinin-M was evaluated after treatment for 48 h with an olive pomace extract (OPT3, 0.10–0.40 mg/mL) and its major compound, hydroxytyrosol (25–100 μM). Regarding the DED animal model, 100 μM hydroxytyrosol, 0.20 mg/mL OPT3, or vehicle (borate buffer) were topically administered to 14 days-desiccating stress-exposed (constant airflow/scopolamine administration) C57BL/6 mice. Tear volume, corneal fluorescein staining (CFS), CD4+, and CD8+ T cell count in lymph nodes (flow cytometry), and *IP-10* and *TNF-α* gene expression (qRT-PCR) in the cornea, conjunctiva, and lacrimal glands were evaluated. OPT3 (0.2–0.4 mg/mL) and hydroxytyrosol (100 μM) significantly reduced hTCD4+ proliferation. In mice, both treatments reduced lacrimal gland *IP-10* gene expression. OPT3 also decreased CFS, and conjunctival *IP-10* and corneal *TNF-α* gene expression. In lymph nodes, hydroxytyrosol reduced CD3+, OPT3, and CD8+ count. Thus, a high-value application as a promising DED protection was proposed for olive pomace.

## 1. Introduction

Dry eye disease (DED) is a very common disorder, with a considerably high prevalence (ranging from 5% to 50% depending on the population) [1] and has a big impact on the quality of life of the patients [2]. A remarkable economic burden is caused by DED, not only by direct costs (medical or treatment) but also by indirect ones (e.g., loss of work productivity) which form the biggest proportion [2]. Immune-mediated inflammation has been considered the principal pathophysiological mechanism for DED, leading to lacrimal functional unit dysfunction, tear fluid alterations, and epitheliopathy in cornea and conjunctiva [3]. Further, elevated inflammatory cell infiltration (e.g., CD4+ T cells) in the conjunctiva and lacrimal gland has also been observed [4], being significantly related to increased tear cytokine/chemokine levels [5].

Mild cases of DED are usually treated with artificial tears, while the treatment approaches for more severe DED are currently based on anti-inflammatory drugs such as topical corticosteroids, antibiotics administration, serum eye drops, or immunosuppressants, such as cyclosporine A [6]. However, side effects have been reported for most of these treatments, such as a strong burning sensation during application or infections related to long-term use [7,8]. Additionally, most corticosteroids cannot be used long-term, as elevation of intraocular pressure and cataract formation may occur [9]. Therefore, their use is restricted and there is still a scarcity of effective therapy. Cyclosporine A is one of the few drugs approved for the indication of DED treatment. It is a calcineurin inhibitor, modifying T cell response by inhibiting Interleukin (IL)-2 activation. It increases tear production, visual acuity, and goblet cell density, while it decreases the damage of the ocular surface epithelium and levels of inflammatory biomarkers in tear film [10,11]. However, in many countries such as Australia and China, cyclosporine A eye drops are not commercially available or can be administered only to more severe DED cases [12,13], while in Europe cyclosporine A is only approved for severe keratitis [12]. In addition, it has very low solubility in water and, therefore, oil or surfactants-based formulations are produced, which usually cause irritations, visual problems, etc. [8,14] Another treatment available is lifitegrast, which acts by inhibiting intercellular adhesion molecule-1, a molecule expressed in DED inflamed epithelium and T cells [15]. Although it was found to be effective in improving the eye dryness score and ocular discomfort, no effect in corneal staining was reported after 84 days of treatment. Additionally, side effects similar to cyclosporine A were observed, including instillation site burning and reaction, decreased visual acuity, and dysgeusia [16], while Xiidra (lifitegrast ophthalmic solution 5.0%) is only available in the US [17,18].

Nowadays, there is increasing interest in the use of natural phenolic compounds as promising therapeutic agents, especially in multifactorial diseases (such as DED), due to their versatile biological activities, including neuroprotective, anticancer, anti-microbial, anti-inflammatory, and antioxidant effects, among others [19]. They are normally consumed in the regular diet through fruits and vegetables and can have considerable potential applicability in the pharmaceutical industry [19,20]. More recently, their use as a treatment for ocular surface inflammatory diseases was proposed based on their anti-inflammatory and immunomodulatory activities [21,22]. Several phenolic compounds, such as quercetin and resveratrol [23,24] or epigallocatechin gallate [25,26], have been proven to be a potential DED therapy based on promising in vitro and in vivo results.

Among the most highlighted phenolic compounds, olive polyphenols are increasingly receiving attention due to their numerous biological activities reported, such as anti-inflammatory, antioxidant, antiviral, and cardioprotective effects, among others [27]. These compounds are present not only in olive oil but also in olive pomace (OP) [28,29]. In fact, during olive oil production, only 1%–2% of the polyphenols of the olive fruit end up in olive oil, as the remaining 98% remain in OP [30]. OP is the solid by-product, a mix of olive pulp, skin, and pit, generated after crushing and spinning out the olive oil during olive oil production [31]. It is produced in Mediterranean countries, mainly in Spain, Italy, Greece, Tunisia, and Turkey, followed by Morocco, Algeria, and Portugal [32]. It is the principal by-product and is produced in huge quantities (between 7 and 30 million m^3^ per year only in the Mediterranean area) [33]. Its high organic/phenolic load increases its biological and chemical demand for oxygen, which together with its phytotoxic properties and storage in open-air spaces generates a major environmental concern, being a relevant pollutant of atmosphere, water, and soil [34,35]. Hence, there is increasing awareness worldwide for the valorization of this by-product, including biotechnological transformations into biofuel or biogas, use as fertilizers on soils, or applications in the composting industry [36], as well as applications in food and cosmetic industries [20]. However, its valorization as a source of high-value phenolic bioactive compounds is of utmost importance for a sustainable development of the olive industry, as the removal of phenolic compounds from OP would offer a by-product more easily processed, with less oxygen demand and, thus, less environmental impact [35,36,37].

Hydroxytyrosol (HT) is one of the most representative olive phenolic compounds [38]. It is a simple phenol, mainly found in olive oil [39], fruit [40], and by-products (OP [41], mill wastewaters [42], and leaves [43]), but also in red and white wines [44]. HT has attracted remarkable scientific interest due to its anti-inflammatory, antiatherogenic, cardioprotective, chemoprotective, antioxidant, and antimicrobial activities [27]. It has also been found to protect from neovascular age-related macular degeneration, diabetic retinopathy, and retinal apoptosis [45,46,47].

The effect of HT and extracts derived from olive by-products on the maintenance of the ocular surface has also been reported. The anti-inflammatory and antioxidant activity of the olive mill wastewaters on rabbit corneal epithelial cells has already been proven [48]. More recently, our group demonstrated the strong antioxidant and anti-inflammatory effect of OP-derived extracts, together with their major compound HT, on human corneal and conjunctival epithelial cells [49]. Thus, the purpose of the present study was to investigate the anti-inflammatory and immunomodulatory activity of HT and a selected OP extract enriched in HT (OPT3), for the protection of DED, in vitro on human CD4+ T cells (hTCD4+) and in vivo in a well-characterized desiccating stress mouse model [50,51].

## 2. Materials and Methods

### 2.1. Plant Material and Extract Preparation

OP of a 2018 crop was kindly provided by Oliduero (Medina del Campo, Spain). It was the solid by-product generated during the olive oil production using the Arbequina variety of olives, which is mostly grown in Spain, but also in California, Argentina, Australia, Chile, and Azerbaijan [52]. Storage conditions and characterization of the material, as well as the extraction procedure and the extract (OPT3) characterization, are all described in detail elsewhere [41]. The extraction conditions were based on the same study, which determined the selective optimal extraction conditions using freeze-dried OP defatted with supercritical carbon CO_2_. Three different optimal extracts (OPT) were produced by pressurized liquid extraction at a distinct percentage of ethanol (EtOH, non-denaturalized-purity: 99.9%, Dávila Villalobos S.L., Valladolid, Spain) in water, temperature, and solid/liquid ratio. OPT3 was produced using a hydroalcoholic solvent with 90.0% *v/v* of EtOH and a solid/liquid ratio of 0.8 g_RAW OP_/mL_SOLVENT_ at 184.0 °C and 10 MPa under N_2_ atmosphere (99.996% purity from Linde Gas, Puçol, Valencia, Spain). An extraction time of 20 min was applied, using freeze-dried OP defatted with supercritical carbon CO_2_ (CO_2_ purity: 99.95%—Carburos Metálicos, Barcelona, Spain). The conditions were selected to produce an extract with the maximum concentration in HT (7.7 ± 0.7 mg/g of dry extract).

### 2.2. In Vitro Immunosuppressive Effect

#### 2.2.1. Cell Isolation and Culture

hTCD4+ cells were isolated from the peripheral blood of healthy volunteers. This study followed the Tenets of the Declaration of Helsinki, and the protocol was approved by the Ethics Committee of the University of Valladolid. Briefly, up to 9 mL of peripheral blood was obtained from 3 healthy male donors with an average age of 28 ± 3 years old, after signing informed consent. Peripheral blood mononuclear cells (PBMCs) were isolated by Ficoll–Paque PLUS density (GE Healthcare, Chicago, IL, USA) gradient centrifugation at 400× *g* for 30 min at room temperature. PBMCs were collected, washed with Dulbecco’s phosphate-buffered saline (DPBS—Thermo Fisher Scientific, Rockford, IL, USA), and used to isolate hTCD4+ cells. hTCD4+ cells were isolated from PBMCs by magnetic separation using a QuadroMACS™ separator (Miltenyi Biotech, Bergisch Gladbach, North Rhine-Westphalia, Germany). Untouched hTCD4+ cells were negatively selected using the CD4+ T Cell Isolation Kit human and LS columns (Miltenyi Biotech, Bergisch Gladbach, North Rhine-Westphalia, Germany), following the manufacturer’s protocol. Isolated hTCD4+ cells were cultured in Roswell Park Memorial Institute (RPMI) 1640 + L-glutamine cell culture medium (Gibco, Grand Island, NY, USA) supplemented with 10% fetal bovine serum (FBS—Thermo Fisher Scientific, Rockford, IL, USA) and penicillin/streptomycin (Thermo Fisher Scientific, Rockford, IL, USA).

#### 2.2.2. Preparation of Phenolic Solutions

HT (≥98% purity, Extrasynthese, Genay, France) and OPT3 were dissolved in DPBS at concentrations of 400 μM (0.062 mg/mL) and 1.6 mg/mL respectively, aliquoted in tubes, and stored at −20 °C. On the day of the experiment, an aliquot of HT or OPT3 was left at room temperature until thawing, and then diluted with cell culture medium to reach the final desired concentrations: 25–100 μM (0.004–0.015 mg/mL) for HT and 0.1–0.4 mg/mL for OPT3. At the end of each day, the remaining quantity was discarded to ensure that no degradation occurred in the compounds [53].

#### 2.2.3. hTCD4+ Cell Proliferation Inhibition Study

hTCD4+ cells were seeded in 96-well plates at a density of 10^5^ cells/well. All cells were activated with 1% phytohemagglutinin—M form (PHA-M—GIBCO, Grand Island, NY, USA), except for the inactivated control. Then, cells were treated for 48 h with 0.1, 0.2, and 0.4 mg/mL of OPT3, and 25, 50, and 100 μM (0.004, 0.008, and 0.015 mg/mL, respectively) of HT. After that time, 10% alamarBlue HS Cell Viability reagent (Invitrogen, Waltham, MA, USA) was added to each well and incubated for 3 h before reading fluorescence at 560 nm excitation and 590 emission wavelengths on a Spectra Max M5 spectrophotometer (Molecular Devices Corporation, Sunnyvale, CA, USA). Three independent experiments were performed in duplicates.

### 2.3. Desiccating Stress (DS) Mouse Model

#### 2.3.1. Animals and DS Conditions

All animal experiments followed the University of Cologne regulations, the German animal protection law (LANUV), and the Association for Research in Vision and Ophthalmology (ARVO) statement for the use of animals in ophthalmic and vision research. They were also approved by the Decentral animal facility (EURL 2010/63) of the Medical Faculty of the University of Cologne.

Female C57BL/6 mice, 10–12 weeks old, were obtained from Charles River Laboratories (Sulzfeld, Germany) and screened for ocular abnormalities (lid edema, corneal opacity, scarring, etc.). DS protocol was applied as previously described [50,51]. Briefly, mice were exposed to DS for 14 days by placing the cages in a controlled environment chamber (conditions: humidity 30 ± 5%, forced airflow 18 h/day, and temperature 25 ± 1 °C). Scopolamine hydrobromide (Sigma-Aldrich, St. Louis, MO, USA) was administered continuously (0.1 mg/day) by subcutaneous implanted osmotic pumps (Alzet, Cupertino, CA, USA). The experiments ended on day 14 and regional lymph nodes, whole eyes with conjunctiva, as well as lacrimal glands were dissected and processed for further analysis as described below. Two experimental sets of 14 days each were performed.

#### 2.3.2. Topical Treatments

HT (100 μM—0.015 mg/mL—Biomol, Hamburg, Germany) and OPT3 (0.2 mg/mL) were dissolved in borate buffer (vehicle-pH = 8.2, Sigma-Aldrich, St. Louis, MO, USA) at the aforementioned concentrations, aliquoted in tubes, and stored at −20 °C. Each treatment day, an aliquot of HT or OPT3 was used and then discarded to ensure that no degradation occurred in the compounds [53]. Mice were distributed randomly into three groups (n = 5 mice/group): (1) HT, (2) OPT3, and (3) vehicle (control). All groups were exposed to the same DS and housing conditions. The treatments started the same day with the setup of the DS (day 1) and were administered topically three times a day (8 AM, 12 AM, and 4 PM) in both eyes (5 μL/eye).

#### 2.3.3. Assessment of Clinical DED Signs

Corneal damage (corneal fluorescein staining—CFS) and tear volume were assessed at baseline—day 0 and day 11 of the DS, as previously described [51]. To evaluate corneal damage, fluorescein staining was used, applying 5 μL/eye of 5% fluorescein sodium salt in DPBS (Thermo F isher Scientific, Rockford, IL, USA). The solution was carefully wiped off after 1 min and grading under blue light was performed with a stereomicroscope. A modified Oxford grading scheme was used with severities varying from grade 0 to 5 [54]. For tear volume, a phenol red thread (Zone Quick Thread—Oasis Medical, San Dimas, CA, USA) was placed in the lateral cantus of each eye for 10 s and a change of color from yellow to red in the wet part (absorption of tears) was measured in millimeters.

#### 2.3.4. Flow Cytometry Analysis (FACS): % Count of CD3+, CD4+ and CD8+ in Lymph Nodes

Lymph nodes of all mice in each of the 3 groups (vehicle, HT, and OPT3-treated) were removed from all animals on the last day and mashed the same day through a cell strainer, as previously described [55]. Briefly, single-cell suspensions were transferred to FACS buffer (0.5% N,O-bis (trimethylsilyl)acetamide—BSA, 1% FBS, and Ethylenediaminetetraacetic acid—EDTA 1:50 in DPBS—Thermo Fisher Scientific, Rockford, IL, USA). After blocking with 0.5 mg/mL Fc block anti-mouse CD16/32 (eBioscience, San Diego, CA, USA) for 15 min, the samples were stained with fluorescent-labelled anti-mouse CD3, CD4, and CD8a antibodies (Table 1—also including their concentrations), for 30 min at 4 °C and protected from light, following the manufacturer’s instructions. Subsequently, cells were washed and resuspended in DPBS, and eFluor450fixable viability dye staining (eBioscience, San Diego, CA, USA) was performed. After washing the cells again, a stabilizing fixative (eBioscience, San Diego, CA, USA) was added. Stained samples were analyzed on a FACS Canto (BD, Germany) and results were extracted using FlowJo Software (FlowJo LLC, Tree Star Inbc., Ashland, OR, USA).

#### 2.3.5. Cytokine/Chemokine Gene Expression

*Ιnterferon γ-induced protein (IP)-10* (also known as CXCL10) and *tumor necrosis factor (TNF)-α* gene expression in the cornea, conjunctiva, and lacrimal gland tissues was studied by quantitative real-time polymerase chain reaction with retrotranscription (qRT-PCR). All procedures were performed as previously described [55]. The corneas and conjunctivas of two eyes per mouse were pooled in the same Eppendorf tube, while for lacrimal glands one sample per mouse was used. All samples were placed in RLT buffer (Qiagen, Hilden, Germany), with 10 μL/mL β-mercaptoethanol (Sigma-Aldrich, St. Louis, MO, USA) and the isolation of the RNA was performed using a RNeasy Plus Mini Kit (Qiagen, Hilden, Germany), according to the manufacturer’s instructions. Reverse transcription was performed using a Revert Aid First-Stand cDNA Synthesis Kit (Thermo Fisher Scientific, Rockford, IL, USA), following the manufacturer’s instructions. To quantify the transcripts of *IP-10* and *TNF-α*, an SYBR Green-based qRT-PCR was performed on the cDNA samples. For the qRT-PCR reactions, a 20 μL volume was used with 20 ng cDNA and 0.75 μM of each forward and reverse primer (Thermo Fisher Scientific, Rockford, IL, USA), together with SsoFast EvaGreen Supermix (Bio-Rad, Bonn, Germany). The incubation included 2 min at 95 °C followed by 45 cycles of 5 sec at 95 °C and 30 sec at 60 °C, using a CFX96 Touch Real-Time PCR Detection System (Bio-Rad, Bonn, Germany). Results of *IP-10* and *TNF-α* were calculated by the comparative threshold method using the hypoxanthine-guanine phosphoribosyl-transferase (HPRT—Thermo Fisher Scientific, Rockford, IL, USA) as housekeeping gene. All samples were performed in triplicates, and water control as well as primer controls were included to detect possible contamination. The primers were designed according to the bibliography [56,57]. Table 2 presents the primer sequences together with their annealing temperatures.

### 2.4. Statistical Analysis

In vitro hTCD4+ cell proliferation data are presented as mean of fold change with respect to PHA-M-activated cells ± standard error of the mean (SEM). Results of CFS score, tear volume (in millimeters), and % IL gene expression in the cornea, conjunctiva, and lacrimal glands are presented as minimum to maximum value in a box and whiskers diagram. Results of % CD3+, CD4+, or CD8+ T cell count in lymph nodes (FACS analysis) are presented as mean of % count ± SEM.

Normality tests were performed on all data, testing their Gaussian distribution using the Kolmogorov–Smirnov test. Based on the results, analysis of variances (ANOVA) or non-parametric test (Kruskal–Wallis) were performed between the groups to analyze statistically significant differences. Two-tailed *p*-values lower than 0.05 were considered statistically significant. For the statistical analyses, the SPSS software (SPSS 15.0; SPSS, Inc., Chicago, IL, USA) was used.

## 3. Results

### 3.1. In Vitro Effect of HT and OPT3 on hTCD4+ Cell Proliferation

**Figure 1:**
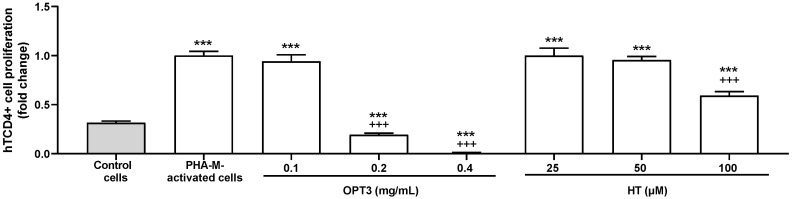
Effect of a selected crude OP extract (OPT3) and HT on the proliferation of PHA-M–activated hTCD4+. Cells were activated with 1% PHA-M, except for the control, and treated for 48 h with OPT3 (0.1–0.4 mg/mL), HT (25–100 μM/0.004–0.015 mg/mL) or vehicle (cell culture medium). Cell proliferation was measured with alamarBlue HS assay (n = 3). *** *p*-value < 0.001, compared with control (non-stimulated) cells; +++ *p*-value < 0.001, compared with vehicle-treated-PHA-M-activated cells.

PHA-M-activated hTCD4+ cells isolated from PBMCs were used to test the immunosuppressive activity of the OPT3 extract and its major compound, HT. The proliferation of hTCD4+ cells was significantly stimulated at 48h by 1% PHA-M (*p*-value < 0.001) (Figure 1). Both treatments significantly reduced PHA-M-activated hTCD4+ cell proliferation at 48 h (Figure 1). In particular, 0.2 mg/mL of OPT3 decreased it by 81 ± 3% (*p*-value < 0.001), 0.4 mg/mL of OPT3 by 99 ± 5% (*p*-value < 0.001), and 100 µM of HT by 41 ± 2% (*p*-value < 0.001). For OPT3, the reduction in cell proliferation at 0.2 and 0.4 mg/mL was also significant compared with the basal levels (control/non-activated cells).

### 3.2. Effect of HT and OPT3 on the DS-Induced DED Animal Model

#### 3.2.1. CFS

**Figure 2:**
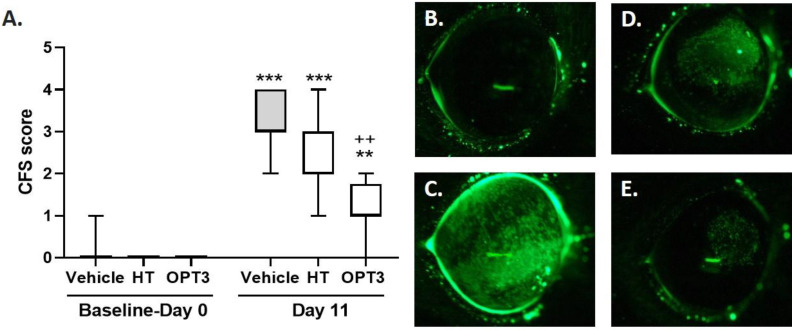
Effect of a selected crude OP extract (OPT3) (0.20 mg/mL) and HT (100 μM/0.015 mg/mL), on CFS score of DS-exposed mice after topical application. (**A**) Corneal damage: *** *p*-value < 0.001, ** *p*-value < 0.01, compared with baseline—day 0 intragroup values, ++ *p*-value < 0.01, compared with vehicle-treated mice (borate buffer) at day 11 of the DS. Representative photos of the murine cornea, in which the epithelial damage is stained in green for: (**B**) Baseline—day 0 grading, (**C**) Vehicle group after DS, (**D**) HT group after DS, and (**E**) OPT3 group after DS. Score grading varied from 0 to 5, depending on the extent of corneal alterations.

After 11 days of DS, all groups demonstrated a statistically significant increase in CFS score (*p*-value < 0.001 in the case of vehicle and HT groups, and *p*-value = 0.0063 for OPT3 group) compared with the baseline—day 0 (Figure 2A). The topical application of OPT3 led to a significant decrease in CFS (*p*-value = 0.0028) of DS-exposed mice, compared with the vehicle-treated group. In the OPT3 group, the maximum CFS score observed was 2, being in some cases even 0 (no staining, no corneal alterations). However, the reduction of CFS score in HT-treated mice compared to the vehicle-treated group was not statistically significant (*p*-value > 0.9999). The difference in CFS score between HT and OPT3-treated groups after 11 days of DS was not significant either (*p*-value = 0.2453).

#### 3.2.2. Tear Volume

**Figure 3:**
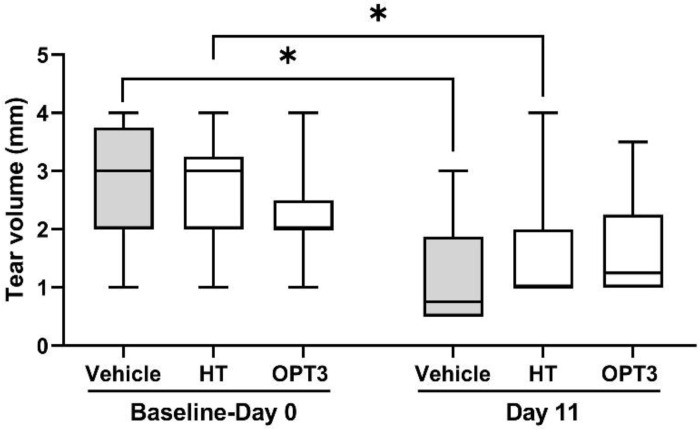
Effect of a selected crude OP extract (OPT3) (0.20 mg/mL) and HT (100 μM/0.015 mg/mL), on tear volume of DS-exposed mice after topical application. * *p*-value < 0.05.

DS and scopolamine administration caused a significant decrease in tear volume compared with baseline levels (*p*-value < 0.05 for both the vehicle and HT group) (Figure 3). For the OPT3 group, the reduction of tear volume was not statistically significant. However, tear volume was not increased by any of the treatments significantly after 11 days of topical administration under DS conditions. No statistically significant differences were observed in tear volume between HT and OPT3-treated groups.

#### 3.2.3. FACS Analysis: % Count of CD3+, CD4+ and CD8+ in Cervical Lymph Nodes

**Figure 4:**
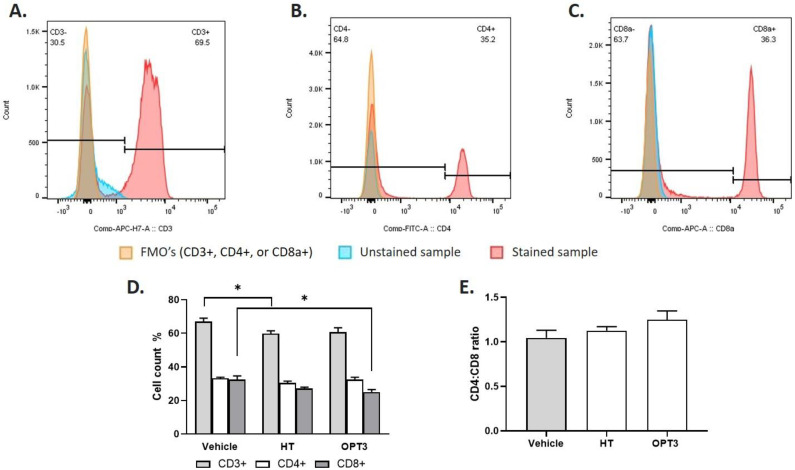
FACS analysis of CD3+, CD4+, and CD8+ T cells of draining lymph nodes of mice exposed to DS for 14 days and received topical treatment of a selected crude OP extract (OPT3) (0.20 mg/mL) and HT (100 μM/0.015 mg/mL). (**A**) Representative FACS histograms for CD3+, (**B**) representative FACS histograms for CD4+, (**C**) Representative FACS histograms for CD8+, (**D**) Percentages of CD3+, CD4+, and CD8+ T cells in terms of total live cells. HT significantly decreased the total number of CD3+, and OPT3 the total number of CD8+, compared with control (vehicle-treated mice). (**E**) Calculated CD4:CD8 ratio. * *p*-value < 0.05.

The number of T cells (CD3, CD4, and CD8) was analyzed in the regional lymph nodes at the end of each experimental set, comparing the vehicle-treated with the HT- and OPT3-treated groups (Figure 4). The scatter properties and gating strategies used for immunophenotyping the cells in the lymph nodes are also presented in the same figure (Figure 4A–C). Regarding the total number of CD3+ lymphocytes (Figure 4D), HT demonstrated a significant decrease compared with the vehicle-treated group (*p*-value = 0.0436), while OPT3 did not show any significant effect (*p*-value = 0.1174). Regarding the percentage of CD8+ T cells (Figure 4D), a significant reduction was observed by OPT3 (*p*-value = 0.0397). CD8+ reduction by HT was not considered statistically significant (*p*-value = 0.3497) compared with vehicle-treated mice. In terms of total CD4+ number (Figure 4D) and CD4:CD8 ratio (Figure 4E), no significant variations were detected between the vehicle-treated group and the treatment groups (neither for HT nor for OPT3). However, for the CD4:CD8 ratio, a tendency for decrease was observed for OPT3 compared with the vehicle group (*p*-value = 0.0944). The differences in CD3+, CD4+, and CD8+ count between HT and OPT3-treated animals were not significant in any of the cases (*p*-value = 0.9532 for CD3+, *p*-value = 0.5137 for CD4+, and *p*-value = 0.9532 for CD8a+) (Figure 4D).

#### 3.2.4. IP-10 and TNF-α Gene Expression in Cornea, Conjunctiva, and Lacrimal Glands

**Figure 5:**
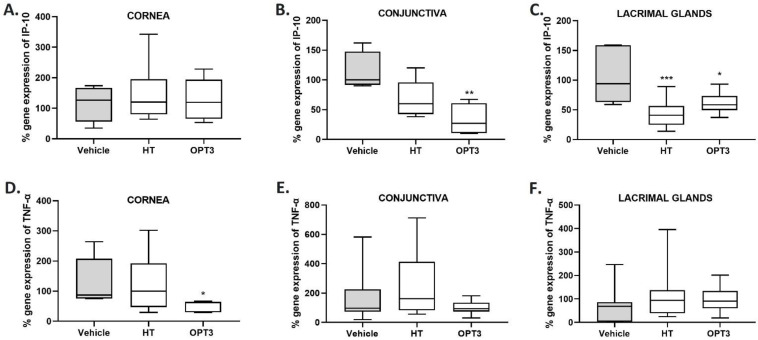
Effect of a selected crude OP extract (OPT3) (0.20 mg/mL) and HT (100 μM/0.015 mg/mL), on *IP-10* (**A**–**C**) and *TNF-α* (**D**–**F**) gene expression in the cornea (**A**,**D**), conjunctiva (**B**,**E**), and lacrimal glands (**C**,**F**) of 14-days-DS-exposed mice after topical application. *IP-10* gene expression was reduced in conjunctiva by OPT3 (**B**) and in lacrimal glands by both treatments (**C**). *TNF-α* gene expression was decreased only in the cornea by OPT3 (**D**). *** *p*-value < 0.001, ** *p*-value < 0.01, * *p*-value < 0.05, compared with vehicle-treated mice (borate buffer).

Gene expression of *IP-10* and *TNF-α* was analyzed in corneal, conjunctival, and lacrimal gland tissues from naïve and DS-exposed mice. A reduction in the expression of these genes related to DED was observed in all tissues by either both or one of the treatments, compared with the control (vehicle-treated) group (Figure 5). % *IP-10* gene expression (Figure 5A–C) was significantly decreased by OPT3 in the conjunctiva (*p*-value < 0.001) and by both treatments in the lacrimal glands (*p*-value < 0.01 for HT, *p*-value < 0.05 for OPT3), compared with vehicle-treated mice. In the conjunctiva, there was also a trend for decrease of *IP-10* expression by HT (*p*-value = 0.0741) (Figure 5B). In the cornea, none of the treatments affected it significantly. Regarding % *TNF-α* gene expression (Figure 5D–F), it was significantly reduced (*p*-value < 0.05) by OPT3 in the cornea, while no significant changes were observed in the conjunctiva and lacrimal glands neither by OPT3 nor by HT. Comparing HT to the OPT3 treated-group, no statistically significant differences were observed either for % *IP-10* gene expression in the conjunctiva (*p*-value = 0.2686) and lacrimal glands (*p*-value = 0.3609) or % *TNF-α* gene expression in the cornea (*p*-value = 0.2016).

## 4. Discussion

This work proposed the use of an agro-industrial by-product that is potentially hazardous for the environment as a potential treatment for DED. The results presented prove that a crude OP extract enriched in HT, namely OPT3, and its major phenolic compound HT, can inhibit hTCD4+ proliferation and reduce clinical signs in a DED animal model. The results from the present work are also in agreement with previous results of our group, in which strong in vitro antioxidant and anti-inflammatory activities of OPT3 and HT were shown on human corneal and conjunctival epithelial cells [49].

Immunomodulatory and anti-inflammatory effects of plant phenolic compounds have widely been studied, including HT and other principal olive compounds [58,59]. In our study, OPT3 and HT were able to significantly decrease in vitro the PHA-M-activated hTCD4+ cell proliferation. The tested concentrations were selected based on the maximum allowable concentration of both treatments on human ocular surface epithelial cells [49]. The pathophysiological mechanism of inflammation of DED has been demonstrated to be immune-mediated and is highly related to the activation and increase of CD4+ and also Th17 T cells [60,61]. Activated T cells are the inflammatory mediators at the ocular surface, reinforcing the damage of goblet cell and epithelial cell loss and leading to ocular surface epitheliopathy and tear film instability [62]. They have been found in the conjunctiva in experimental DED studies [63], and also in patients with DED and Sjögren’s syndrome [64,65]. In addition, the severity of conjunctiva inflammation depends on T cell activation [65].

Mechanistically, for HT, a high affinity with the CD4 cellular receptor has been shown, e.g., preventing human immunodeficiency virus (HIV) from bonding during viral infection [66]. The anti-inflammatory effect of HT observed in this study is comparable to that observed by other natural phenolic compounds such as isorhamnetin, curcumin, resveratrol, and vanillic acid, which reduced the secretion of pro-inflammatory cytokines in vitro on Jurkat CD4+ T cells, also suggesting a synergistic activity on cytokine modulation [67]. Resveratrol has also been found to inhibit CD4+ activation in vitro and in vivo [68], while cirsilineol, another phenolic compound, was proven to be a potential treatment for T-cell-mediated inflammatory bowel diseases due to its targeted activity on CD4+ cells [69]. In addition, enzymatically polymerized polyphenols derived from caffeic acid, *p*-coumaric acid, and ferulic acid have been found to bind with a recombinant CD4 protein and CD4 molecules on the cell surface [70].

In our study, the protective and anti-inflammatory effect of OPT3 and HT on the ocular surface was also demonstrated in vivo for the first time. The doses of HT (100 μM–0.015 mg/mL) and OPT3 (0.2 mg/mL) were selected based on our previous in vitro experimental work [49], the current in vitro results on hTCD4+, and the existing bibliography of same or similar compounds/extracts [46,71]. OPT3 dramatically decreased the CFS score in DS-exposed mice. These results demonstrated that topical application of OPT3 improves corneal integrity in mice exposed to DS and, thus, protects the ocular surface. The protective effect of other polyphenols on corneal integrity has already been studied. Abengózar-Vela et al. [23] proved that 0.1 mg/mL of quercetin and a mixture of 0.1 mg/mL quercetin with 1 mg/mL resveratrol reduced the CFS score when applied topically in a similar DED animal model. Epigallocatechin gallate also decreased CFS in vivo either as a pure solution at 0.1 mg/mL and 1 mg/mL after four and nine days of topical application, respectively [26], or formulated in gelatin-g-poly (N-isopropylacrylamide) copolymers after three days of treatment [72]. Similarly, catechin in a fourteen days DED rabbit model demonstrated the reversion of corneal damage [73]. Topical application of phenolic extracts was also found to reduce CFS, i.e., 1 mg/mL of a mixture of ethanolic extracts from different medicinal plants (*Schizonepeta tenuifolia*, *Angelica dahurica, Rehmanniag lutinosa Liboschitz, Makino*, and *Cassia tora* L) after ten days of treatment [74] and 1 mg/mL of *Chamaecyparis obtusa* extract after seven days of treatment in mouse models of DED [75]. However, in our study, not only was there an improvement in the corneal integrity, but also the valorization of an agro-industrial by-product. This study also aimed to add extra value to the related industries and contribute to the emerging solutions to environmental pollution. It should be highlighted that at the concentration tested (0.20 mg/mL), OPT3 comprised 0.0015 mg/mL (10.0 μM) of HT (i.e., 10 times less compared with the pure HT tested). Thus, there is a strong synergistic effect in the OP extract. Although no statistically significant difference was detected between HT and OPT3-treated groups, OPT3 was able to reduce CFS, whereas HT did not. This can probably be attributed to the synergistic activity of the phenolic compounds present in it.

Our results also showed that OPT3 and HT did not affect tear volume. Apart from the exposure to DS conditions, this can be attributed to the subcutaneous scopolamine administration, which blocks the already shown muscarinic activity of polyphenols [76]. In accordance with our results, quercetin and resveratrol (alone or in combination) did not improve tear volume either after topical administration in DS-exposed mice [23]. In contrast, while using the same DS-model as in our study (including the use of scopolamine), Oh et al. [77] presented increases in tear volume after quercetin topical administration, probably because of the different types of mice used and the different concentrations and daily application times. Thus, more experiments are needed to evaluate the effect of the treatments on lacrimal glands and tears. However, as previously described, corneal integrity was maintained by OPT3, even when the tear film was not restored. Thus, this treatment can be effective in the case of aqueous deficient DED (in which lacrimal secretion is decreased in conditions of normal evaporation of the tear fluid from the eye), probably by protecting the cornea [62].

In accordance with the in vitro results on hTCD4+ cells, the in vivo data also indicate the modulatory effect of OPT3 and HT on immune cells and their possible preventive effect on lymph nodes in the early phase of the DED animal model. The important role of T cells for the development and progression of the DED has already been described [64,65]. Activated T cells are highly infiltrated into the lacrimal functional unit tissues due to DS conditions [3]. Cyclosporine A, a T cell inhibitor, acts on T cells by suppressing their lymphokine (IL-2) secretion and is proven to be highly effective regarding symptoms and signs in DED patients [78]. Lymph nodes are the reservoir of immune cells and are the place where adaptive immunity is regulated and autoreactive CD4+ and CD8+ T cells are activated [79]. Apart from CD4+, a significant increase of CD8+ has also been observed for draining lymph nodes of DS-exposed mice [80]. Therefore, in this study, the effect of the OP phenolic compounds on the CD4+ and CD8+ ratio in the lymph nodes was studied. This effect was comparable to another study, where a significant decrease of CD4+ T cell infiltration was observed in the conjunctiva of recipient nude mice, after being adoptively transferred with CD4+ isolated from resveratrol- and quercetin-treated mice exposed to DS for ten days, compared with vehicle-treated mice [23]. However, since we did not study any marker of activation in the isolated T cells, nor did we study the effect of adoptively transfer them to naive nude mice, we cannot discard that our results were in part related to T cell trafficking. Thus, more detailed studies are needed to fully conclude the effect of HT and OPT3 on cervical lymph nodes T cell activation and on the local tissues and lacrimal glands.

We also demonstrated that both HT and OPT3 phenolic treatments significantly reduced the gene expression of cytokines/chemokines in the lacrimal functional unit tissues. In particular, OPT3 reduced *TNF-α* gene expression in the cornea and *IP-10* gene expression in the conjunctiva and lacrimal glands, while HT decreased *IP-10* gene expression in the lacrimal glands. Increased levels of these molecules have been related to the pathophysiology of the DED and have been observed in conjunctiva and tears of DED patients [5,81]. High levels of *IP-10* and increased *TNF-α* transcripts were also detected in the corneal and conjunctival epithelium of mice exposed to DS [82,83]. Previous in vitro data from our group already demonstrated the strong inhibition of *IP-10* secretion by OPT3 and HT on human corneal and conjunctival epithelial cells [49]. In accordance with our results, the reduction of levels or expression of cytokines/chemokines related to the DED in the lacrimal functional unit of a DED animal model has already been reported for phenolic compounds or phenolic-rich extracts. Topical application of several polyphenols has been proved to significantly downregulate the cytokine levels in the cornea (0.2 mg/mL of epigallocatechin gallate [84]) and lacrimal glands (30 mg/mL of 7-carboxymethyloxy-3′,4′,5-trimethoxy flavone [85]) of DED rabbits, as well as lacrimal glands (10 mg/mL of catechin [86]) and tear fluid (0.1 mg/mL quercetin and 1 mg/mL resveratrol—alone or in combination [23]) of DED mice. In addition, topical application of phenolic-rich extracts in the conjunctiva of DS-induced mice inhibited the expression (1 mg/mL of a mixture of ethanolic extracts of pharmaceutical plants: *Schizonepeta tenuifolia* var. *japonica Kitagawa*, *Angelica dahurica* Bentham et Hooker, *Rehmannia glutinosa Liboschitz* var. *purpurea*, *Makino*, and *Cassia tora* L. [74]) and the levels (0.1 mg/mL of a *Chamaecyparis obtusa* leaves extract [75] and 1 mg/mL of *Camellia japonica* leaves extract [87]) of several cytokines. Further studies addressing the effect of HT and OPT3 in other DE-relevant cytokines (such IL-6, MMP-9 or IL-17A among others) expression and/or secretion are warranted.

This work has some limitations that should be addressed in future studies. First, the application of the treatments was performed in parallel with the development of the DED under DS conditions and scopolamine administration, demonstrating that our treatments are effective in inhibiting the development of the disease. As patients visit the ophthalmologist with already established symptoms, additional studies starting the treatment after the fourteen days of DS conditions would explore the possible treating capacity and downregulating effect of the olive phenolic compounds on established DED conditions. Second, only one concentration of HT and OPT3 was tested, demonstrating strong protective immunomodulatory and anti-inflammatory effects; however, more concentrations should be tested to detect any further possible effectiveness, but also safety issues. Third, since a clear synergistic effect was detected for OPT3 compared with the pure HT; different OP-derived phenolic compounds (e.g., oleacein, hydroxytyrosol acetate, quinic acid, etc.) present in the OPT3 extract [53] or with good prior in vitro results (e.g., oleuropein) [49] should be tested as a pure solution, alone or in combination with HT, to identify more potential bio-active olive secondary metabolites. Lastly, regarding the in vitro results, both treatments demonstrated a strong immunosuppressive effect on hTCD4+ cells; however, their effect on the cytokine/chemokine secretion by hTCD4+ should also be examined.

## 5. Conclusions

This work demonstrated that a selected extract derived from OP and its major phenolic compound, HT, can provide promising topical protection for the DED, despite the limitations. Both treatments not only demonstrated an immunosuppressive effect in vitro on hTCD4+ cells but also reduced the inflammatory response (cytokine gene expression) in the lacrimal functional unit of a DED animal model. Additionally, OPT3 reduced the in vivo clinical signs (corneal damage) of dry eye. Thus, this study demonstrated the translational relevance of the OP phenolic compounds as a promising future DED treatment. Additionally, this work is paramount for the sustainable development of olive industries, proposing a high-value alternative use of an environmentally hazardous agro-industrial by-product as therapy for immune-based ocular surface diseases. Additionally, the results of the present study prove the efficacy of the phenolic compounds for DED and warrantee more tests in the future, including clinical studies about their safe use in the healthy ocular surface to ensure their use as DED treatment.

## 6. Patents

The results are patent pending.

## Figures and Tables

**Table 1:** Antibody panel used for FACS analysis.

Antibody (Clone)	Target	Conjugation	Manufacturer	Catalog No.	Concentration (mg/mL)
Anti-mouse CD3 (17A2)	CD3+ T cells	allophycocyanin-cyanine 7 (APC-Cy7)	Biolegend (San Diego, CA, USA)	100221	0.10
Anti-mouse CD4 (GK1.5)	CD4+ T cells	fluorescein isothiocyanate (FITC)		100405	0.125
Anti-mouse CD8a (53–6.7)	CD8+ T cells	APC		100711	0.05

**Table 2:** Selected primers for qRT-PCR.

mRNA	Sequence	Annealing Temperature
HPRT	F: 5′-TTGGATACAGGCCAGACTTTGTTG-3′ R: 5′-GATTCAACTTGCGCTCATCTTAGGC-3′	60 °C
IP-10	F: 5′-ATATACGCGTTGACATTGATTATTGACTAG-3′ R: 5′-ATTGCTAG-CAGCTGGTTCTTTCCGCCTC-3′	60 °C
TNF-α	F: 5′- AGGACTCAAATGGGCTTTCC-3′ R: 5′-CAGAGGCAACCTGACCACTC-3′	63 °C

F = forward primer, R = reverse primer, IP-10 = interferon γ-induced protein-10, TNF-α = Tumor Necrosis Factor-α, HPRT = hypoxianthine-guanine phosphoribosyl-transferase.

## Data Availability

Not applicable.

## Abbreviations

APC	Allophycocyanin
APC-Cy7	Allophycocyanin-cyanine7
BSA	N,O-bis(trimethylsilyl)acetamide
CFS	Corneal fluorescein staining
DED	Dry eye disease
DPBS	Dulbecco’s phosphate buffered saline
DS	Desiccating stress
EDTA	Ethylenediaminetetraacetic acid
EtOH	Ethanol
FACS	Flow cytometry analysis
FBS	Fetal bovine serum
FITC	Fluorescein isothiocyanate
HPRT	Hypoxanthine-guanine phosphoribosyl-transferase
HIV	Human immunodeficiency virus
HT	Hydroxytyrosol
hTCD4+	human CD4+ T cells
IL	Interleukin
IP-10	Interferon γ-induced protein-10
OP	Olive pomace
OPT3	Selected crude olive pomace extract
PBMC	Peripheral blood mononuclear cells
PHA-M	Phytohemagglutinin—M form
qRT-PCR	Quantitative real-time polymerase chain reaction
RPMI	Roswell Park Memorial Institute
SEM	Standard error of the mean
TNF-α	Tumor necrosis factor-α
